# The Contractile Phenotype of Skeletal Muscle in TRPV1 Knockout Mice Is Gender-Specific and Exercise-Dependent

**DOI:** 10.3390/life10100233

**Published:** 2020-10-06

**Authors:** Aude Lafoux, Sabine Lotteau, Corinne Huchet, Sylvie Ducreux

**Affiliations:** 1 Therassay Platform, CAPACITES, Université de Nantes, 44200 Nantes, France; aude.lafoux@univ-nantes.fr; 2 CarMeN Laboratory, University of Lyon, INSERM, INRA, INSA Lyon, Université Claude Bernard Lyon 1, 69500 Bron, France; sabine.lotteau@gmail.com; 3 Nantes Gene Therapy Laboratory, INSERM UMR 1089, Université de Nantes, 44200 Nantes, France; Corinne.huchet2@univ-nantes.fr; 4 Département de Cardiologie, Hospices Civils de Lyon, Groupement Hospitalier EST, IHU-OPERA Bâtiment B13, 69500 Bron, France

**Keywords:** TRPV1, skeletal muscle, exercise

## Abstract

The transient receptor potential vanilloid 1 (TRPV1) belongs to the transient receptor potential superfamily of sensory receptors. TRPV1 is a non-selective cation channel permeable to Ca^2+^ that is capable of detecting noxious heat temperature and acidosis. In skeletal muscles, TRPV1 operates as a reticular Ca^2+^-leak channel and several *TRPV1* mutations have been associated with two muscle disorders: malignant hyperthermia (MH) and exertional heat stroke (EHS). Although TRPV1^−/−^ mice have been available since the 2000s, TRPV1’s role in muscle physiology has not been thoroughly studied. Therefore, the focus of this work was to characterize the contractile phenotype of skeletal muscles of TRPV1-deficient mice at rest and after four weeks of exercise. As MS and EHS have a higher incidence in men than in women, we also investigated sex-related phenotype differences. Our results indicated that, without exercise, TRPV1^−/−^ mice improved in vivo muscle strength with an impairment of skeletal muscle in vitro twitch features, i.e., delayed contraction and relaxation. Additionally, exercise appeared detrimental to TRPV1^−/−^ slow-twitch muscles, especially in female animals.

## 1. Introduction

Transient receptor potential (TRP) channels form the ion channel family with the greatest variety of selectivity and activation mechanisms [1]. Among TRPs, transient receptor potential vanilloid 1 (TRPV1) was the first family member to be cloned [2] and to have its structure revealed [3]. TRPV1 is a nonselective cation channel with a very high Ca^2+^ permeability (P_Ca_/P_Na_ ~ 10) [2]. TRPV1 was originally described as the capsaicin receptor responsible for the sensation of spiciness upon ingestion of hot chili peppers. Since its discovery, TRPV1 has been implicated in nociception, inflammation, and many other biologic processes [4,5,6]. Since heat and acidosis [7,8] are both physiological parameters modified during muscle contraction and exercise, they can activate TRPV1, and thus it is clear that TRPV1 is a key player in muscle physiology.

Research progress on TRPV1 function was facilitated by the creation of a TRPV1^−/−^ mouse model 20 years ago [9]. Since then, numerous articles have focused on phenotypic modifications for these transgenic animals, yet only a few have considered sex-related differences, mostly focusing on nociception [10,11]. Among this literature, contradictory results have been reported for the TRPV1^−/−^ spontaneous locomotor activity, ranging from increases in the light or in the dark phase to decreases in both phases, depending on the experimental setup used and the animal age (for a review, see [12]). Likewise, TRPV1’s impact on physical performance remains unclear. Several works have demonstrated that TRPV1 activation via oral administration of capsaicinoids could improve endurance capacity in rodents [13,14,15], whereas others have illustrated that TRPV1 desensitization did not impair swimming performance [16]. To date, contractile function of TRPV1^−/−^ skeletal muscles has not been fully studied. At the cellular level, we and others have found that TRPV1 channels are expressed mainly in the sarcoplasmic reticulum (SR) and involved in Ca^2+^ homeostasis [17,18,19]. Lately, we divulged a genetic link between *TRPV1* mutations and two muscle pathologies, namely malignant hyperthermia (MH) and exertional heat stroke (EHS) [20,21,22].

MH and EHS are the two main triggered hyperthermia types regarded as phenotypes of a latent skeletal muscle disorder, as they share several hallmarks [23,24,25,26]. First, both clinically present a dysregulation of muscle contraction, leading to an increase in body temperature and potentially to multiorgan failure. Acute crises can be, respectively, elicited by volatile anesthetics or strenuous sport activities (MH incidence: 1/5000–1/50,000 anesthetized patients; EHS incidence: 1/500 runners) and are more common in men than in women [27,28]. Without treatment, MH and EHS can lead to patient death. Second, both diseases are linked to mutations of proteins involved in the sarcoplasmic reticulum Ca^2+^ release; most are related to the ryanodine-receptor type 1 gene *(RYR1*) [26,29,30], to which are now added the *TRPV1* mutations. Our previous reports proposed that TRPV1-induced SR Ca^2+^ release could provoke SR Ca^2+^ release via ryanodine receptors 1 and that TRPV1 mutated channels could shape this crosstalk [19,20,22]. Third, existing treatments, involving the use of dantrolene for MH or rapid cooling for EHS [31,32], aim to stop the crises but not prevent them. Most patients are asymptomatic in their daily life. However, with the incredibly elevated number of athletes participating in marathons, trails, and extreme sports, it has become urgent to elucidate the precise mechanisms that lead to pathologic hyperthermia.

In the present work, we primarily aimed to appraise the contractile phenotype of TRPV1^−/−^ skeletal muscles and to investigate whether sex-based differences existed; therefore, we evaluated 12 to 16-week-old male and female wild-type (WT) and TRPV1^−/−^ mice. The mice were submitted to an increase in physical activity with a 4-week treadmill protocol to better understand the role of TRPV1 in muscle functions. A thorough knowledge of TRPV1’s role in skeletal muscle is essential for developing new therapeutic strategies against skeletal muscle diseases as MH or EHS.

## 2. Results

### 2.1. Genotype-Related Differences Were Found in Body Weight and in the Relative Heart, Diaphragm, and Extensor Digitorum Longus Weights, but not in Tibialis Anterior and Soleus Muscles

**Figure 1:**
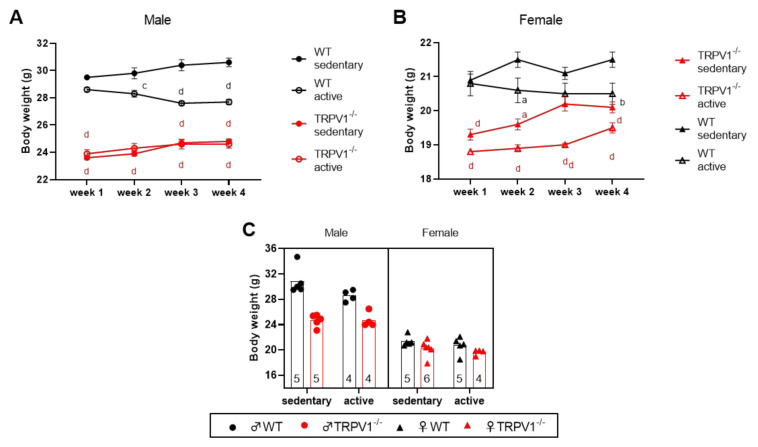
Body weight in the different mouse groups. (**A**,**B**) Body weight was measured weekly in (**A**) males and (**B**) females. Data are means ± standard error of the mean (SEM; *n =* 4–6). ^a^ *p* < 0.05, ^b^ *p* < 0.01, ^c^ *p* < 0.001, ^d^ *p* < 0.0001 (two-way ANOVA) compared to the control WT sedentary group; (**C**) scatter plots of body weight at the end of the treadmill protocol. Sedentary animals (left) and (right) active animals. Black symbols—wild-type (WT) mice; red symbols—TRPV1^−/−^ mice; circles—males; triangles—females; plain symbols—sedentary animals; open symbols—active animals. Sample sizes appear within bars (bottom). Three-way ANOVA results presented in Table S1.

To examine how different TRPV1-genotype mice respond to exercise, we used a moderate treadmill training protocol for three sessions of 30 min exercise per week. In this study, 12-week-old male and female mice were trained (or not) for four weeks and weighed weekly. At the beginning of the training protocol, TRPV1-deficient mice were already lighter (male: 23.75 ± 1.91 g; female: 19.05 ± 3.18 g) than their wild-type (WT) counterparts (male: 29.05 ± 5.18 g; female: 20.85 ± 0.64 g) (Figure 1A,B). While WT animals lost weight as the training progressed, the TRPV1^−/−^ animals slightly gained body weight. At the end of the increased physical activity period, a three-way ANOVA was performed to test three factors: genotype (WT vs. TRPV1^−/−^), sex (male vs. female), and exercise (sedentary vs. active). The results presented in Table S1 indicated a significant effect of exercise, sex, genotype, and sex–genotype interaction on mouse body weight (Figure 1C). Active mice were significantly (~4.8%) lighter than sedentary mice, with male mice being ~32% heavier than female mice. TRPV1^−/−^ mice were also lighter than WT mice, but not in the same range in male (~15.3%) and female (~3%) mice.

**Figure 2:**
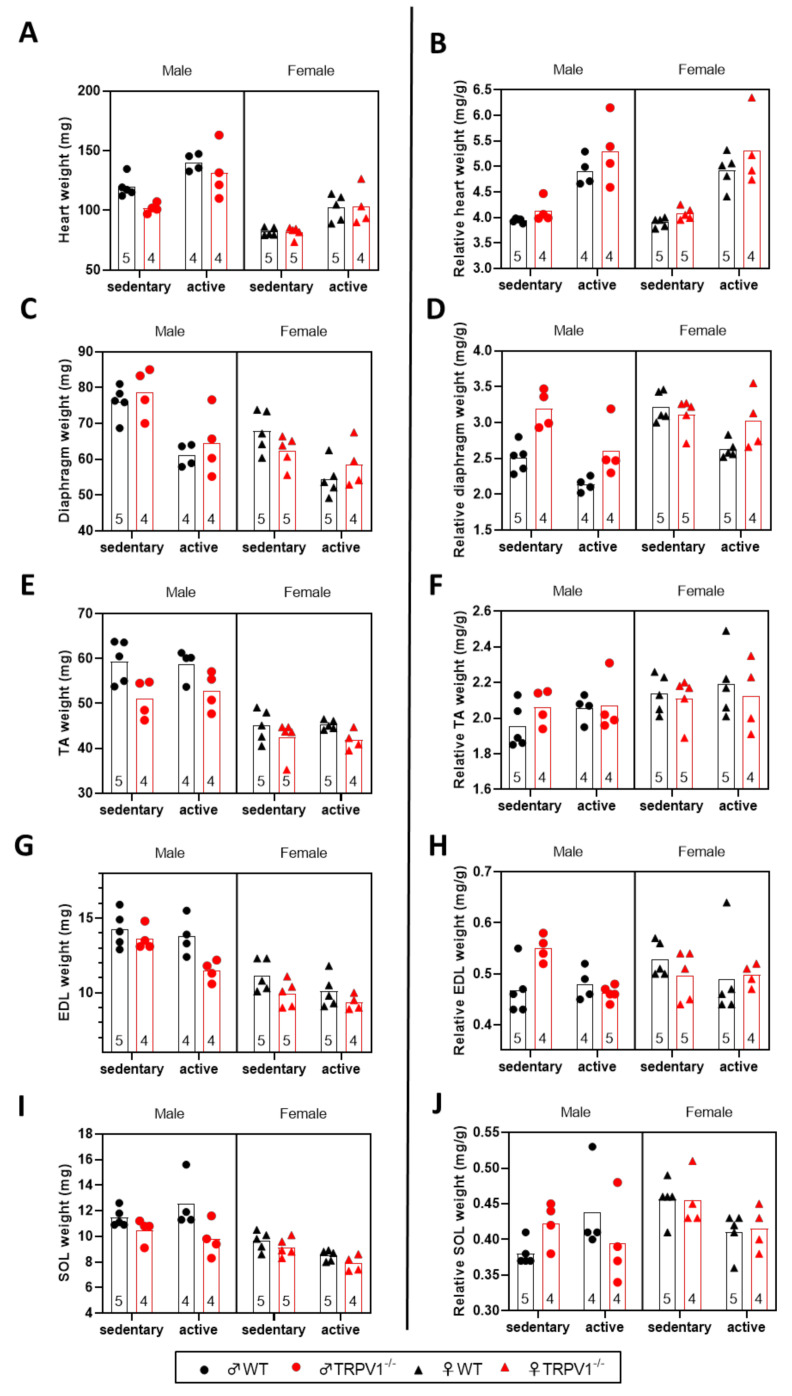
Absolute and relative muscle weights into the different mouse groups. Scatter plots of the (**A**,**C**,**E**,**G**,**I**) absolute and (**B**,**D**,**F**,**H**,**J**) relative weights of (**A**,**B**) heart, (**C**,**D**) diaphragm, (TA; **E**,**F**) tibialis anterior, (EDL; **G**,**H**) extensor digitorum longus and (SOL; **I**,**J**) soleus muscles. Sedentary animals (left) and (right) active animals. Black symbols—WT mice; red symbols—TRPV1^−/−^ mice; circles—males; triangles—females. Relative weights normalized to body weight (mg/g). Bars represent means. Sample sizes appear within bars (bottom). Three-way ANOVA results presented in Tables S1–S4.

Figure 2A displays absolute values of heart weight that were higher in males (~+33.5%) than in females and higher in active animals (~+23.8%) than in sedentary ones (Table S1). When normalized to body weight, heart weight was significantly (~27.4%) greater in the active mice than in sedentary ones (Figure 2B). There was also a significant difference between the two genotypes; the ratio of heart weight to body weight was significantly (~6.4%) higher in TRPV1^−/−^ mice compared to their WT counterparts. No sex-related differences and no interaction between the three factors were observed (Table S2).

The absolute weights of the diaphragms were greater in male animals (~+15.3% than females) and in sedentary animals (~+19.4% than active animals; Figure 2C; Table S1). The relative diaphragm muscle weight was significantly lowered by ~13.5% in the active group compared to sedentary ones. It was significantly raised by ~13.6% in TRPV1^−/−^ animals in comparison to WT animals and by ~14.6% in females in comparison to males (Figure 2C). As for body weight, a significant sex-by-genotype interaction was detected (Table S2); the relative diaphragm weight was superior for TRPV1^−/−^ males than for other animals.

We next examined three skeletal muscles: the tibialis anterior (TA; Figure 2D,E), the extensor digitorum longus (EDL; Figure 2G,H), and the soleus (SOL; Figure 2I,J) (Tables S3 and S4). The absolute weights of the three muscles were more important in males and in WT mice, but only the EDL absolute weights were found to be less developed because of the training program (~–8.4%). Concerning the relative values, sex-related diversity appeared for the TA weight in a statistically different manner: female relative weights were ~5.2% higher than males, and this sex-specific effect was only marked when female sex was associated with exercise for SOL muscles. In parallel, the relative loss in EDL weight was more important in the active TRPV1^−/−^ male group than in any other active groups. Because of the observed weight variations, we chose to present only the relative values rather than the absolute values in the rest of the work.

### 2.2. Exercise, Female Sex, and TRPV1 Deficiency Improved Muscle Strength

**Figure 3:**
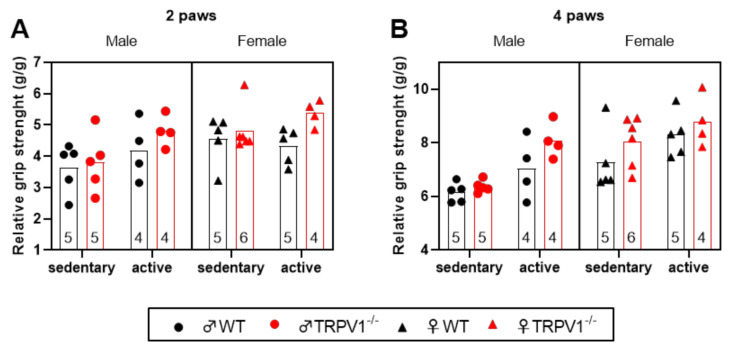
Grip strength tests into the different mouse groups. Scatter plots of the relative maximal muscle strength of (**A**) forelimbs (2 paws) and (**B**) combined forelimbs and hind limbs (4 paws). Sedentary animals (left) and (right) active animals. Black symbols—WT mice; red symbols—TRPV1^−/−^ mice; circles—males; triangles—females. Results normalized to body weight (g/g). Bars represent means. Sample sizes appear within bars (bottom). Three-way ANOVA results presented in Table S5.

Thereafter, we used the grip strength test to evaluate the neuromuscular function as maximal muscle strength of forelimbs (two-paw) and combined forelimbs and hind limbs (four-paw). As illustrated in Figure 3 and in Table S5, the three-way ANOVA tests revealed that exercise significantly increased the relative maximal strength by ~15.8% in the four-paw test (~12.1% in two-paw; *p =* 0.0562). Female mice showed significantly improved strength by ~16.1% and 17% compared to male mice, respectively, in two-paw and four-paw tests. Additionally, TRPV1^−/−^ mice showed a significant elevation in the relative maximal strength of ~12.1% and 9%, respectively, in the two- and four-paw tests. We saw no interaction between the three tested factors.

### 2.3. TRPV1 Deficiency Modified the Contractile Properties of Skeletal Muscles in a Muscle Type-Specific Manner

**Figure 4:**
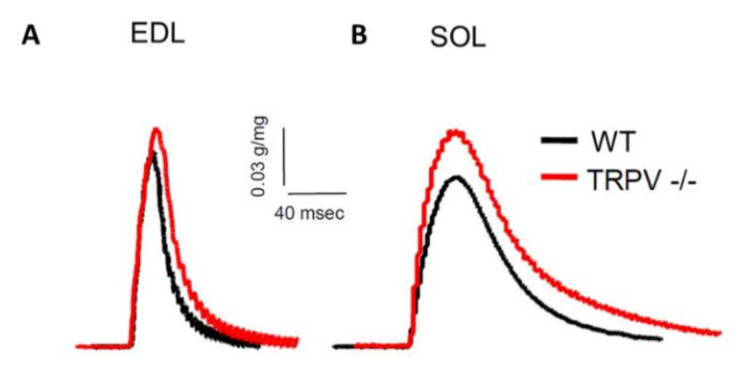
Contractile properties of isolated slow- and fast-twitch skeletal muscles in male mice under sedentary conditions. Representative twitch traces of extensor digitorum longus (EDL) and soleus (SOL) muscles from sedentary male wild-type (WT, black lines) and TRPV1^−/−^ (TRPV^−/−^, red lines) mice. Twitches elicited by a single stimulation 0.5 ms duration and six-volt amplitude at room temperature.

**Figure 5:**
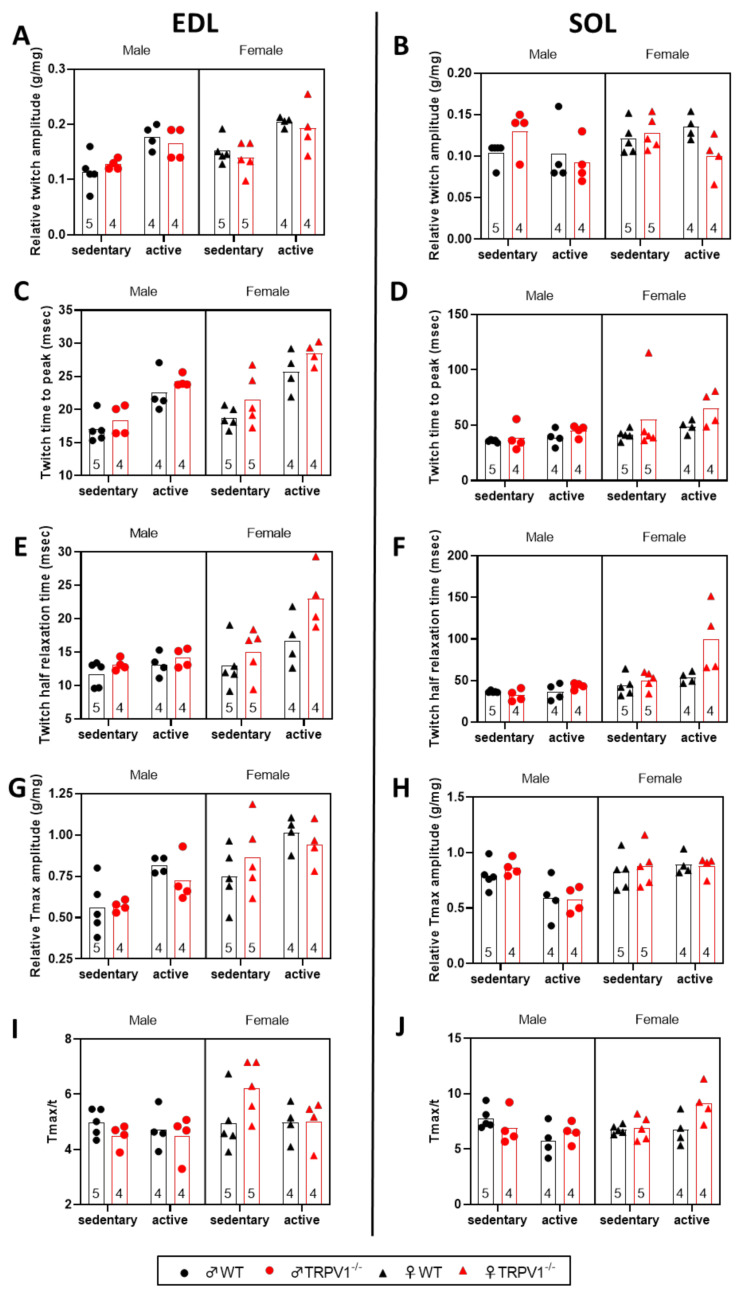
In vitro comparison of twitch and tetanus parameters of extensor digitorum longus (EDL) and soleus (SOL) muscles into the different mouse groups. Scatter plots of the (**A**,**B**) relative peak tension, (**C**,**D**) time-to-peak tension, (**E**,**F**) half-relaxation time, (Tmax; **G**,**H**) relative tetanic force and (Tmax/t; **I**,**J**) twitch/tetanus ratio in (**A**,**C**,**E**,**G**,**I**) EDL fast-twitch muscle, and (**B**,**D**,**F**,**H**,**J**) SOL slow-twitch muscle. Sedentary animals (left) and (right) active animals. Black symbols—WT mice; red symbols—TRPV1^−/−^ mice; circles—males; triangles—females. Results normalized to the muscle weight (g/mg). Bars represent means. Sample sizes appear within bars (bottom). Three-way ANOVA results presented in Tables S6–S8.

In the following, we wanted to identify the differences in contractile properties exhibited by the different mice in EDL fast-twitch muscles and in SOL slow-twitch muscles (Figure 4 and Figure 5, Tables S6–S8). Figure 4 illustrates typical twitch responses obtained from in situ muscle contraction measurements in sedentary males.

As expected, exercise increased the relative twitch amplitude to muscle weight ratio and the twitch kinetics (time to peak and half relaxation time) of EDL muscles by ~38.5%, 33.3%, and 26.9%, respectively (Figure 5A,C,E; Table S6). Female EDL muscles demonstrated larger relative twitch amplitude and slower kinetics parameters. Although TRPV1 deficiency did not influence the relative EDL twitch tension, it delayed both the time to peak and the half-time relaxation time by ~10.3% and 19.7%, respectively. No factor interaction was observed except for an exercise-by-sex interaction on the half relaxation time; EDL muscles of active females required longer to relax.

When we similarly analyzed the SOL twitch parameters (Figure 5B,D,F; Table S7), the relative twitch tension was found to be only influenced by exercise-by-genotype interaction, with active TRPV1 mice showing a weaker twitch tension than other mice. Males had significantly faster twitch kinetics than females. SOL contraction in TRPV1^−/−^ mice was slightly delayed by ~23.6% *(p =* 0.0716) and SOL relaxation was significantly retarded by ~32.8% in comparison to WT muscles. Notably, the combination of two by two factors, exercise/female, exercise/TRPV1-genotype, or female/TRPV1-genotype, delayed the relaxation of SOL muscles.

The relative tetanus amplitude (Figure 5F,G; Table S8) of the fast- and slow-twitch muscles was amplified by the exercise or by the female sex, but independent of mouse genotype. The tetanus-to-twitch ratio was significantly greater (~13.3%) in female than in male EDL muscles. This ratio was augmented in SOL muscles by the following combinations: exercise/female and exercise/TRPV1-genotype (Figure 5I,J; Table S8).

### 2.4. Force–Voltage and Force–Frequency Relationships Were Disturbed in Slow-Twitch Muscles but Not in Fast-Twitch Muscles by the TRPV1 Deficiency

**Figure 6:**
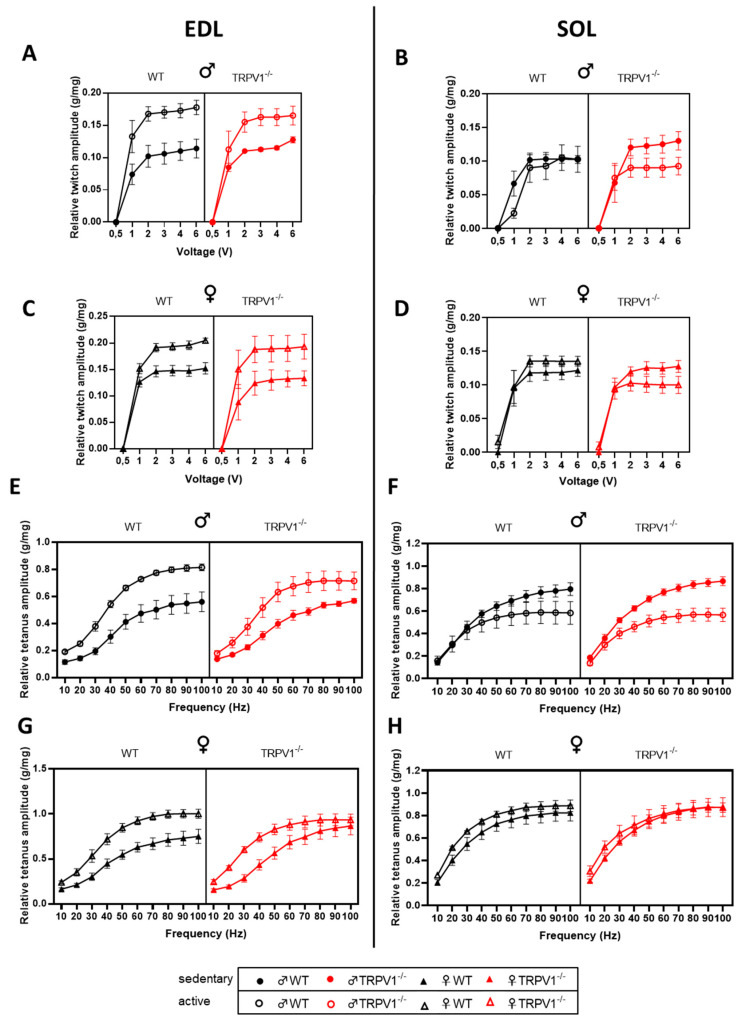
Force–voltage and force–frequency relationships of extensor digitorum longus (EDL) and soleus (SOL) muscles into the different mouse groups. (**A**,**C**,**E**,**G**) In vitro EDL and (**B**,**D**,**F**,**H**) SOL relative muscle (**A**–**D**) force–voltage and (**E**–**H**) force–frequency relationships in (**A**,**B**,**E**,**F**) male and (**C**,**D**,**G**,**H**) female mice. Black symbols—WT mice; red symbols—TRPV1^−/−^ mice; circles—males, triangles—females; plain symbols—sedentary animals; open symbols—active animals. Results presented as means ± SEM *(n =* 4–5) and normalized to muscle weight (g/mg). Three-way ANOVA results presented in Tables S9 and S10.

To further evaluate the degree of fiber recruitment, we analyzed the relationship between stimulus strength (0.5 to 6 V) and the relative twitch force (Figure 6). The force was developed by the increase in physical activity in male and female fast-twitch muscles across genotypes. For the slow-twitch muscles, the results are more complex: in males, exercise had a negative influence on the muscle force of WT (on average ~–13.7%) and TRPV1^−/−^ (on average ~–21.6%) animals. In female animals, the combined effect of TRPV1-deficiency and exercise was deleterious on the slow-twitch muscle force (on average ~–15.8%) whereas the performance of WT active mice slightly ameliorated (on average ~+11.6%; Figure 6A–D; Table S9).

The frequency dependence of contraction was used to indicate the efficiency of the excitation–contraction coupling process. As the frequency increased, the force was improved by the training protocol in male and female EDL muscles across genotypes. In SOL muscles, the force–frequency relationship was negatively shaped by exercise in male mice and almost unchanged in female mice, and these dissimilarities were intensified in TRPV1-deficient mice (Figure 6E–H; Table S10).

### 2.5. TRPV1 Deficiency Did Not Alter Fatigue Profiles of Fast- and Slow-Twitch Muscles

**Figure 7:**
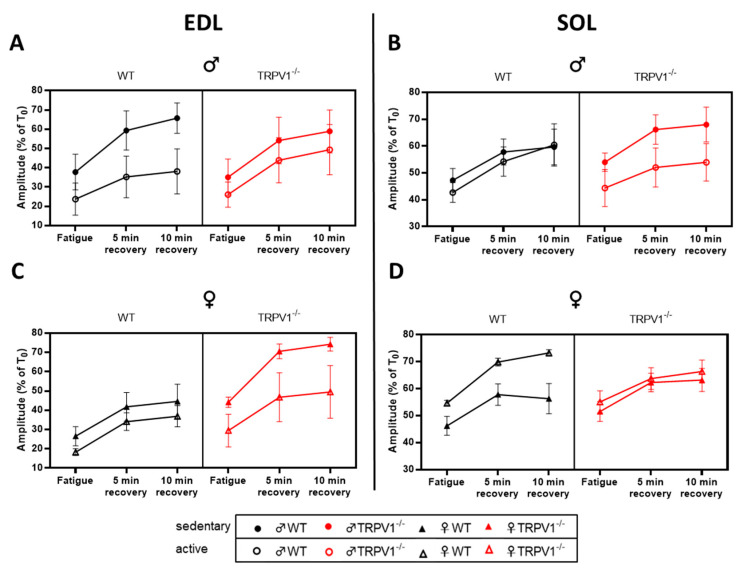
Fatigue of EDL and SOL muscles into the different mouse groups. In vitro EDL (**A,C**) and SOL (**B,D**) relative tetanus amplitude in the fatigue protocol obtained in male (**A,B**) and female (**C,D**) mice at fatigue, and 5 and 10 min recovery times. Black symbols—WT mice; red symbols—TRPV1^−/−^ mice; circles—males; triangles—females; plain symbols—sedentary animals; open symbols—active animals. Results presented as means ± SEM *(n =* 4–5) and normalized to relative pre-fatigue tetanus amplitude (T_0_). Three-way ANOVA results presented in Tables S11 and S12.

The two muscle types were subjected to a fatigue protocol consisting of 105 repetitions of tetanic contractions (Figure 7; Tables S11 and S12). The specific force produced by the EDL muscles produced during the whole fatiguing protocol was significantly lower in active mice than sedentary mice, across sex and genotype (Figure 7A and Figure 6C; Table S11). On the contrary, the SOL specific force only diminished in active males but ameliorated in active females in comparison with sedentary animals, as shown by the significant exercise-by-sex interaction of the three-way ANOVA (Figure 7B,D; Table S12). Note that the mouse genotype did not influence the force of the fast- or slow-twitch muscles at fatigue, nor after 5 or 10 min of recovery.

## 3. Discussion

The main conclusions of the present study are: TRPV1^−/−^ mice showed no weight loss due to exercise; TRPV1^−/−^ mice appeared muscularly stronger than their WT counterparts; the impact of TRPV1 deficiency was more visible on slow-twitch muscles than on fast-twitch ones; the combination of TRPV1-deficiency with the female-sex or with exercise (or with both factors) combined emphasized some muscle-specific differences.

As quality control for our study, we found expected results concerning the effects of increased physical activity, sex, or their coordinated effect. Hence, our moderate training protocol affected almost every parameter evaluated in this study, ameliorating the contractile properties of fast-twitch muscles and reducing those of slow-twitch muscles. Our work also highlights well known differences between male and female mice, which have been attributed to the hormonal regulation or myosin chain isoform differences [33,34]. In addition, female mice showed an enhanced exercise capacity as previously reported [35]. Notably, the increase in physical activity, which is accompanied by cardiorespiratory adaptation, led to a reduction in the weight of the diaphragm. Nevertheless, compensation by intercostal muscles, which were not evaluated in this study, cannot be excluded.

Then, we found that transgenic mice were lighter than their wild-type littermates before treadmill activity, as recently observed by Inprasit et al. [36]. In their study, TRPV1^−/−^ female mice maintained their body weight even under eight weeks of high fat diet without changing their relative fat mass. The authors concluded that TRPV1 may contribute to the regulation of body weight. Despite training three times per week, our transgenic mice gained weight, reinforcing and extending their conclusions to both sex.

Returning to our initial question of whether TRPV1^−/−^ mice have a skeletal phenotype, given our results, the answer is positive. In vivo, TRPV1^−/−^ mice demonstrated an improvement in neuromuscular function that we attribute to peripheral TRPV1, since ablation of TRPV1-expressing neurons did not alter the grip strength of WT mice [37]. In all mice groups, maximal muscle strength markedly increased with four weeks of treadmill training. This result supports those of other authors who found that moderate physical activity maintains skeletal muscle function as spontaneous locomotion in mice and in humans [38,39]. In vitro, TRPV1 deficiency globally deteriorated the increase force production of the slow-twitch muscles compared to fast-twitch ones in our experimental conditions. To summarize, TRPV1 absence only affected the twitch kinetics and the relative weight of EDL muscles. Opposite to SOL muscles, the scope of modifications linked to the absence of TRPV1 alone or combined with exercise or sex is broader, including twitch amplitude and kinetics, tetanus properties, and force–voltage and force–frequency relationships. It could not be excluded that the increase in physical activity in control and TRPV1^−/−^ mice induced fiber types conversion in muscles, so additional histological experiments are needed.

As to why the lack of TRPV1 impairs muscle function in a muscle-type specific way, several explanations can be proposed. First, a close link between TRPV1 and PGC-1α, which drives the formation of slow-twitch muscle fibers [40], has been a focus in the literature. Capsaicin activation of TRPV1 enhanced PGC-1α expression in C2C12 myotubes and thereby promoted mitochondrial function and exercise endurance [14]. It would not be surprising if in the absence of TRPV1, PGC-1α expression would be scaled down, explaining the marked effect on slow-twitch muscle performance. Second, TRPV1 could directly affect the mitochondrial function, even if the available data on the subject are antagonistic. TRPV1 activation intensified mitochondrial damage in a cardiac cell line [41] whereas it precluded mitochondrial dysfunction by blocking membrane-associated membranes (MAMs) formation in mouse glomeruli [42]. Another reason could be attributed to expression of different TRPV1 splicing isoforms—a long isoform ubiquitously expressed and a short isoform showing a restricted expression [43]. In a muscle-type-dependent manner, one or several isoforms may have a specific expression profile; TRPM8, another TRP family member, could contribute differently to Ca^2+^ homeostasis [44]. These questions deserve future investigation.

Although TRPV1^−/−^ mouse skeletal muscles have never been phenotyped before our investigation, other members of the TRPV family had. Naticchioni et al. reported that the contractile function of skeletal muscles was unchanged in TRPV2 null mice [45]. Similarly, lack of TRPV4 did not cause any alteration of muscular force production or fatigue [46]. These works have only evaluated on one muscle type: EDL for TRPV2 and SOL for TRPV4. It may be interesting to see whether muscle-type specificity exists as we observed for TRPV1. The literature on the impact of the pharmacological modulation of TRPV1 on muscle function is barely more abundant. Only one study revealed that pharmacological activation of TRPV1 by capsaicin decreases the tension of fast muscles in frogs [47].

As a side finding, we noticed that there was no effect of genotype on fatigue. Presumably, TRPV1 deficiency affects the muscle force without affecting the fiber composition switch.

Our results are relevant with regard to triggered hyperthermia. The gold standard procedure to test the susceptibility for MH is the in vitro contracture test (IVCT). IVCT consists of testing the contracture responses to halothane and caffeine and classifying tested patients into three categories: normal (MHN; negative tests), susceptible (MHS; negative tests), and equivocal (MHE; one out of two positive tests). This test relies on a biopsy in the quadriceps muscles (either vastus medialis or vastus lateralis). Both muscles contain a higher proportion of fast twitch fibers [48,49]. The muscle-type-specific incidence of TRPV1 expression could explain why some patients are classified as equivocal. Similarly, Elbas et al. recently showed that a *RYR* mutation affects only slow-twitch muscle [50]. Given these outcomes, a slow-type muscle should be included in future tests. Some genotype-related differences have been revealed only in association with exercise, reinforcing the link between TRPV1 and EHS. Marathon runners or practitioners of prolonged sport activity have mostly slow-twitch muscle fibers. Heat stroke could appear not only due to the rise in the intramuscular temperature, but also due to the increasing proportion of slow muscles engaged during the effort, two parameters that trigger the activation of TRPV1. Other genotype-related variations appeared together with the female sex in our study. The rate of heat stroke illness (EHI) is higher in women than in men, whereas for EHS, the most severe type of EHI, the frequency of crisis is higher in men than in women [51]. One hypothesis is that the probability of developing triggered hyperthermia relies not only on RyR1 alone or on TRPV1 alone, but rather on a specific RyR1/TRPV1 association. As these channels are heterotetrameric, the possible combinations are numerous.

To conclude, we conducted a functional exploration of the motor function of TRPV1^−/−^ mice. In the absence of TRPV1, slow muscles are less efficient in basal conditions than in active conditions. This specific muscle difference is important to examine in future work to better understand the common mechanisms of MH and EHS.

## 4. Materials and Methods

### 4.1. Animals

C57Bl6 J WT mice were used as controls (Charles River, L’Arbresle, France). TRPV1^−/−^ (B6.129X1-Trpv1^tm1Jul^IJ) mice were bred in our laboratory from two couples bought from the Jackson Laboratory. Experiments were carried out on male and female WT and TRPV1^−/−^ littermates, aged between 12 and 16 weeks *(n =* 4–6 animals per group). Mice were housed 4–5 per cage and maintained on a 12/12 h light/dark schedule in a temperature-controlled facility (22 ± 1 °C) with free access to food and water. Animals were kept undisturbed for 7 days before experiments. All procedures were conducted in conformity with European rules for animal experimentation (French Ethical Committee APAFIS#8186-2016121315485337; date of approval: 10 January 2017).

### 4.2. Treadmill Protocol

Mice were randomly allotted to four groups according to sex and genotype. As previously described [52], half of each group was allotted to some concurrent action program on a treadmill. In the beginning, 12-week-old male and female mice were introduced to the treadmill, which had a Plexiglas^®^ cover to stop their escape and offered a running surface that was 100 cm in length and 7.5 cm in width. Initial 10 min physical activity sessions of slow speed walking (5 m/min) were conducted to familiarize the animals with the equipment. The speed of the treadmill and the length of the session were progressively increased so that mice were able to walk at a speed of 10 m/min for 30 min 3 times per week without enforcement by electrical shock. All the animals well tolerated this exercise program and were categorized as “active mice”. The other half of the mice population was released for the same duration to the immovable treadmill, and they were categorized as “sedentary mice”. This protocol was continued over a 4-week period until the mice reached the age of 16 weeks.

### 4.3. Grip Test

The grip test is a noninvasive method designed to evaluate muscle strength in vivo by taking advantage of the mouse’s tendency to grasp a grid while suspended by its tail. The grip test is then used to assess the maximal muscle strength of the four limbs. Mice were placed on a 10 × 10 cm grid with their forepaws (2 paws) or fore/hind paws (4 paws) onto a grid and were gently pulled backward until they released their grip [53]. A grip meter (Bio-GT3, BIOSEB, Vitrolles, France) attached to a force transducer measured the peak force generated. Three steps were completed and averaged. The results are normalized to the body weight (g/g, relative grip strength).

### 4.4. Contractile Properties of Fast- and Slow-Twitch Muscles

EDL and SOL muscle force properties were analyzed using in situ muscle contraction measurements as described previously [54,55]. Succinctly, mice were anesthetized by intraperitoneal injection of pentobarbital (50 mg/kg) and the adequacy of the anesthesia was monitored throughout the experiment. The skin was then taken out of the left leg and the EDL muscle was dissected intact. The tendon of the EDL muscle was attached to a force transducer. The tibia and the foot were mended by two clamps and positioned parallel to the tibia. Mice were kept on heating pads to maintain body temperature and muscles were perfused with a Ringer solution. Stimulation electrodes were positioned at the middle of the muscle and connected to a pulse generator. The muscle was stretched and the stimulation voltage was adjusted to produce the most powerful twitch contractions. Twitch parameters (tension, time to peak, and half-relaxation time) were measured at different voltages (0.5 ms duration, 0.5–6 V stimulation amplitude, increments of 0.5 V). For each voltage, five contractions were analyzed. After a 3 min pause, tetanic forces were recorded at different frequencies of stimulation (200 ms bursts, 6 V, 10–100 Hz, increments of 10 Hz, 1 burst every 30 s). After a 3 min pause, muscles were exposed to the fatigue protocol (105 repeated 250 ms long tetanic contractions, 60 Hz, 1 every 4 s). Recovery was analyzed after 5 and 10 min rests. All force data are expressed as percentage of the initial force, i.e., the force developed before fatigue protocol. After EDL measurements, a similar protocol was performed for SOL muscle. At the end of the experiments, EDL, SOL, TA, diaphragm muscles, and heart were rapidly dissected and weighed. Twitch and tetanic forces were normalized to grams per milligram of fresh EDL and SOL muscles. Data were collected and stored for analysis with Chart v4.2.3 (PowerLab 4/25 ADInstruments, PHYMEP France).

### 4.5. Statistical Analysis

Two-way ANOVA was used to test the effect of genotype and exercise on body weight. Three-way ANOVA of differences among experimental variants were performed to test the effects of genotype, sex, and exercise, as well as interactions on body weight, relative organ weights, grip strength, and in vitro muscle contraction. Except for tension–voltage and tension–frequency relationships, differences were tested among frequency or voltage, genotype, and exercise. Statistical analysis was performed with GraphPad Prism version 8.0.0 for Windows, GraphPad Software, San Diego, California USA. *p <* 0.05 was considered statistically significant.

## Supplementary Materials

The following are available online at https://www.mdpi.com/2075-1729/10/10/233/s1, Table S1: Results of three-way ANOVA testing the effects of genotype (WT vs. TRPV1^−/−^), sex (male vs. female) and exercise (sedentary vs. trained) on body weight, absolute weights of heart and diaphragm, Table S2: Results of three-way ANOVA testing the effects of genotype (WT vs. TRPV1^−/−^), sex (male vs. female) and exercise (sedentary vs. trained) on relative weights of heart and diaphragm, Table S3: Results of three-way ANOVA testing the effects of genotype (WT vs. TRPV1^−/−^), sex (male vs. female) and exercise (sedentary vs. trained) on absolute weights of TA, EDL and SOL muscles, Table S4: Results of three-way ANOVA testing the effects of genotype (WT vs. TRPV1^−/−^), sex (male vs. female) and exercise (sedentary vs. trained) on relative weights of TA, EDL and SOL muscles, Table S5: Results of three-way ANOVA testing the effects of genotype (WT vs. TRPV1^−/−^), sex (male vs. female) and exercise (sedentary vs. trained) on relative two paws and four paws grip strength tests, Table S6: Results of three-way ANOVA testing the effects of genotype (WT vs. TRPV1^−/−^), sex (male vs. female) and exercise (sedentary vs. trained) on relative twitch amplitude and twitch kinetics of EDL muscles, Table S7: Results of three-way ANOVA testing the effects of genotype (WT vs. TRPV1^−/−^), sex (male vs. female) and exercise (sedentary vs. trained) on relative twitch amplitude and twitch kinetics of SOL muscles, Table S8: Results of three-way ANOVA testing the effects of genotype (WT vs. TRPV1^−/−^), sex (male vs. female) and exercise (sedentary vs. trained) on relative tetanus amplitude (Tmax) and tetanus to twitch ratio (Tmax/t) of EDL and SOL muscles, Table S9: Results of three-way ANOVA testing the effects of voltage of stimulation (from 0.5 V to 6 V), genotype (WT vs. TRPV1^−/−^) and exercise (sedentary vs. trained) on relative force-voltage relationship of EDL and SOL muscles, Table S10: Results of three-way ANOVA testing the effects of frequency of stimulation (from 10 to 100 Hz), genotype (WT vs. TRPV1^−/−^) and exercise (sedentary vs. trained) on relative force-frequency relationship of EDL and SOL muscles, Table S11: Results of three-way ANOVA testing the effects of genotype (WT vs. TRPV1^−/−^), sex (male vs. female) and exercise (sedentary vs. trained) on relative tetanus amplitude at fatigue, 5 min recovery and 10 min recovery times of EDL muscles, Table S12: Results of three-way ANOVA testing the effects of genotype (WT vs. TRPV1^−/−^), sex (male vs. female) and exercise (sedentary vs. trained) on relative tetanus amplitude at fatigue, 5 min recovery and 10 min recovery times of SOL muscles.
