# Supplementary material for: The Contractile Phenotype of Skeletal Muscle in TRPV1 Knockout Mice Is Gender-Specific and Exercise-Dependent
Source: Life (Basel). 2020 Oct 6;10(10):233. doi: 10.3390/life10100233 (PMC7600525; doi:10.3390/life10100233)
Supplement: Supplementary file 1 [file life-10-00233-s001.pdf]

Supplementary Materials

x: interaction of factors; %: percentage of total variation; F: F ratio (DFn: degree of freedom numerator, DFd: degree of freedom denominator). Significant differences (p<0.05) are given in bold.

Table S1. Results of three-way ANOVA testing the effects of genotype (WT vs. TRPV1$^{-/-}$), sex (male vs. female) and exercise (sedentary vs. trained) on body weight, absolute weights of heart and diaphragm.

	Body weight			Absolute heart weight			Absolute diaphragm weight		
**Factor**	**%**	**F (1, 30)**	**P value**	**%**	**F (1, 28)**	**P value**	**%**	**F (1, 28)**	**P value**
**Exercise (E)**	1.14	4.35	**P=0.0455**	26.4	37.3	**P<0.0001**	37.7	35.2	**P<0.0001**
**Sex (S)**	67.9	259	**P<0.0001**	48.2	68.0	**P<0.0001**	24.3	22.6	**P<0.0001**
**Genotype (G)**	14.2	54	**P<0.0001**	2.11	2.97	P=0.0956	0.36	0.34	P=0.5654
**E x S**	0.09	0.33	P=0.5691	0.23	0.33	P=0.5706	2.6	2.43	P=0.1304
**E x G**	0.55	2.09	P=0.1588	0.37	0.522	P=0.4761	1.81	1.69	P=0.2048
**S x G**	5.82	22.2	**P<0.0001**	2.33	3.28	P=0.0807	1	0.93	P=0.3428
**E x S x G**	0.49	1.89	P=0.1799	0.18	0.26	P=0.6162	1.36	1.27	P=0.2690

Table S2. Results of three-way ANOVA testing the effects of genotype (WT vs. TRPV1$^{-/-}$), sex (male vs. female) and exercise (sedentary vs. trained) on relative weights of heart and diaphragm.

	Relative heart weight			Relative diaphragm weight		
**Factor**	**%**	**F (1, 28)**	**P value**	**%**	**F (1, 28)**	**P value**
**Exercise (E)**	69.9	78.47	**P<0.0001**	22.8	22.34	**P<0.0001**
**Sex (S)**	0.02	0.02	P=0.8960	20.3	19.91	**P=0.0001**
**Genotype (G)**	4.60	5.16	**P=0.0311**	17.8	17.47	**P=0.0003**
**E x S**	0.04	0.05	P=0.8286	0.63	0.62	P=0.4391
**E x G**	0.61	0.68	P=0.4165	0.71	0.7	P=0.4112
**S x G**	<0.01	<0.01	P=0.9801	6.45	6.34	**P=0.0178**
**E x S x G**	<0.01	<0.01	P=0.9849	4.23	4.15	P=0.0511

Table S3. Results of three-way ANOVA testing the effects of genotype (WT *vs.* TRPV1$^{-/-}$), sex (male *vs.* female) and exercise (sedentary *vs.* trained) on absolute weights of TA, EDL and SOL muscles.

	**Absolute TA weight**			**Absolute EDL weight**			**Absolute SOL weight**		
**Factor**	**%**	**F (1, 28)**	**P value**	**%**	**F (1, 29)**	**P value**	**%**	**F (1, 27)**	**P value**
**Exercise (E)**	0.02	0.03	P=0.8616	7.03	10.3	**P=0.0033**	2.31	2.20	P=0.1493
**Sex (S)**	64.7	94.1	**P<0.0001**	61.0	89.3	**P<0.0001**	45.8	43.6	**P<0.0001**
**Genotype (G)**	12.0	17.5	**P=0.0003**	9.22	13.5	**P=0.0010**	13.1	12.5	**P=0.0014**
**E x S**	0.07	0.1	P=0.7519	0.36	0.53	P=0.4741	4.26	4.05	P=0.0538
**E x G**	0.07	0.1	P=0.7565	0.57	0.83	P=0.3687	1.98	1.89	P=0.1804
**S x G**	1.99	2.90	P=0.0997	0.34	0.5	P=0.4832	3.78	3.6	P=0.0682
**E x S x G**	0.25	0.36	P=0.5509	1.79	2.63	P=0.1163	1.49	1.42	P=0.2433

Table S4. Results of three-way ANOVA testing the effects of genotype (WT *vs.* TRPV1$^{-/-}$), sex (male *vs.* female) and exercise (sedentary *vs.* trained) on relative weights of TA, EDL and SOL muscles.

	**Relative TA weight**			**Relative EDL weight**			**Relative SOL weight**		
**Factor**	**%**	**F (1, 28)**	**P value**	**%**	**F (1, 29)**	**P value**	**%**	**F (1, 27)**	**P value**
**Exercise (E)**	2.39	0.87	P=0.3597	8.26	3.51	P=0.0713	2.65	1.134	P=0.2964
**Sex (S)**	13.0	4.73	**P=0.0383**	1.73	0.73	P=0.3984	8.61	3.688	P=0.0654
**Genotype (G)**	0.06	0.02	P=0.8863	1.02	0.43	P=0.5161	0.01	0.0058	P=0.9399
**E x S**	0.15	0.05	P=0.8171	1.02	0.43	P=0.5161	11.4	4.865	**P=0.0361**
**E x G**	1.43	0.52	P=0.4767	2.39	1.01	P=0.3223	5.27	2.256	P=0.1447
**S x G**	3.49	1.27	P=0.2700	5.11	2.17	P=0.1516	0.01	0.0058	P=0.9399
**E x S x G**	0.22	0.08	P=0.7771	12.7	5.39	**P=0.0275**	6.99	2.994	P=0.0950

Table S5. Results of three-way ANOVA testing the effects of genotype (WT vs. TRPV1$^{-/-}$), sex (male vs. female) and exercise (sedentary vs. trained) on relative two paws and four paws grip strength tests.

	**Two paws**			**Four paws**		
**Factor**	**%**	**F (1, 30)**	**P value**	**%**	**F (1, 30)**	**P value**
**Exercise (E)**	7.95	3.94	P=0.0562	21.8	15.62	**P=0.0004**
**Sex (S)**	15.3	7.57	**P=0.0100**	26.0	18.63	**P=0.0002**
**Genotype (G)**	9.46	4.69	**P=0.0383**	7.40	5.31	**P=0.0283**
**E x S**	3.32	1.65	P=0.2089	0.85	0.61	P=0.4422
**E x G**	3.31	1.64	P=0.2095	0.29	0.21	P=0.6505
**S x G**	0.64	0.33	P=0.5785	<0.01	<0.01	P=0.9927
**E x S x G**	0.25	0.12	P=0.7280	1.44	1.04	P=0.3168

Table S6. Results of three-way ANOVA testing the effects of genotype (WT *vs.* TRPV1$^{-/-}$), sex (male *vs.* female) and exercise (sedentary *vs.* trained) on relative twitch amplitude and twitch kinetics of EDL muscles.

	**Relative twitch amplitude**			**Time to peak**			**Half relaxation time**		
**Factor**	**%**	**F (1, 27)**	**P value**	**%**	**F (1, 27)**	**P value**	**%**	**F (1, 27)**	**P value**
**Exercise (E)**	45.18	30.25	**P<0.0001**	53.20	53.30	**P<0.0001**	17.44	11.69	**P=0.0020**
**Sex (S)**	11.75	7.87	**P=0.0092**	12.64	12.67	**P=0.0014**	21.09	14.14	**P=0.0008**
**Genotype (G)**	0.56	0.37	P=0.5470	6.28	6.29	**P=0.0185**	10	6.70	**P=0.0153**
**E x S**	0.03	0.02	P=0.8963	0.53	0.53	P=0.4725	7.43	4.98	**P=0.0341**
**E x G**	0.69	0.46	P=0.5029	0.01	0.01	P=0.9090	1.2	0.81	P=0.3770
**S x G**	0.66	0.44	P=0.5129	0.51	0.51	P=0.4831	3.1	2.08	P=0.1612
**E x S x G**	0.74	0.49	P=0.4881	0.01	0.01	P=0.9081	1.88	1.26	P=0.2720

Table S7. Results of three-way ANOVA testing the effects of genotype (WT *vs.* TRPV1$^{-/-}$), sex (male *vs.* female) and exercise (sedentary *vs.* trained) on relative twitch amplitude and twitch kinetics of SOL muscles.

	Relative twitch amplitude			Time to peak			Half relaxation time		
**Factor**	**%**	**F (1, 27)**	**P value**	**%**	**F (1, 27)**	**P value**	**%**	**F (1, 27)**	**P value**
**Exercise (E)**	6.59	2.69	P=0.1129	4.16	1.65	P=0.2103	13.20	10.42	**P=0.0033**
**Sex (S)**	7.23	2.94	P=0.0976	15.81	6.27	**P=0.0186**	26.35	20.81	**P<0.0001**
**Genotype (G)**	0.4	0.16	P=0.6884	8.87	3.52	P=0.0716	8.34	6.59	**P=0.0161**
**E x S**	1.51	0.62	P=0.4397	0.36	0.14	P=0.7077	6.23	4.92	**P=0.0351**
**E x G**	14.21	5.7879	**P=0.0233**	0.2	0.08	P=0.7805	7.03	5.56	**P=0.0259**
**S x G**	4.81	1.96	P=0.1731	2.63	1.04	P=0.3159	6.75	5.33	**P=0.0289**
**E x S x G**	0.07	0.03	P=0.8668	<0.01	<0.01	P=0.9696	2.21	1.74	P=0.1980

Table S8. Results of three-way ANOVA testing the effects of genotype (WT *vs.* TRPV1$^{-/-}$), sex (male *vs.* female) and exercise (sedentary *vs.* trained) on relative tetanus amplitude (Tmax) and tetanus to twitch ratio (Tmax/t) of EDL and SOL muscles.

	EDL						SOL					
	**Relative Tmax**			**Tmax/t**			**Relative Tmax**			**Tmax/t**		
**Factor**	**%**	**F (1, 27)**	**P value**	**%**	**F (1, 27)**	**P value**	**%**	**F (1, 27)**	**P value**	**%**	**F (1, 27)**	**P value**
**Exercise (E)**	22.20	14.29	**P=0.0008**	4.2	1.77	P=0.1944	9.55	4.94	**P=0.0349**	0.05	0.023	P=0.8801
**Sex (S)**	31.79	20.46	**P=0.0001**	12.4	5.23	**P=0.0302**	22.35	11.55	**P=0.0021**	4.77	2.18	P=0.1517
**Genotype (G)**	0.07	0.04	P=0.8355	0.61	0.26	P=0.6170	0.46	0.24	P=0.6291	4.19	1.91	P=0.1784
**E x S**	0.19	0.12	P=0.7298	1.63	0.69	P=0.4145	17.09	8.83	**P=0.0062**	16.77	7.64	**P=0.0101**
**E x G**	3.24	2.09	P=0.1600	1.88	0.79	P=0.3807	1.21	= 0.63	P=0.4353	11.47	5.23	**P=0.0303**
**S x G**	0.64	0.41	P=0.5274	8.04	3.39	P=0.0764	0.03	0.01	P=0.9027	5.43	2.48	P=0.1273
**E x S x G**	0.29	0.19	P=0.6677	4.3	1.81	P=0.1891	0.01	<0.01	P=0.9356	0.37	0.17	P=0.6842

Table S9. Results of three-way ANOVA testing the effects of voltage of stimulation (from 0.5 V to 6 V), genotype (WT *vs.* $TRPV1^{-/-}$) and exercise (sedentary *vs.* trained) on relative force-voltage relationship of EDL and SOL muscles.

	EDL						SOL					
	Male			Female			Male			Female		
**Factor**	**%**	**F (DFn, DFd)**	**P value**	**%**	**F (DFn, DFd)**	**P value**	**%**	**F (DFn, DFd)**	**P value**	**%**	**F (DFn, DFd)**	**P value**
**Voltage (V)**	69.74	F (5, 78) = 71.99	P<0.0001	71.70	F (5, 78) = 67.01	**P<0.0001**	65.96	F (5, 78) = 39.31	**P<0.0001**	72.98	F (5, 84) = 59.14	**P<0.0001**
**Genotype (G)**	0.02	F (1, 78) = 0.08	P=0.7757	0.65	F (1, 78) = 3.05	P=0.0846	0.97	F (1, 78) = 2.9	P=0.0923	1.14	F (1, 84) = 4.61	**P=0.0346**
**Exercise (E)**	12.58	F (1, 78) = 64.93	P<0.0001	9.1	F (1, 78) = 42.49	**P<0.0001**	2.74	F (1, 78) = 8.15	**P=0.0055**	<0.01	F (1, 84) = 0.01	P=0.9194
**V x G**	0.02	F (5, 78) = 0.02	P=0.9998	0.19	F (5, 78) = 0,.17	P=0.9744	0.77	F (5, 78) = 0.46	P=0.8071	0.36	F (5, 84) = 0.29	P=0.9163
**V x E**	2.66	F (5, 78) = 2.74	P=0.0246	1.9	F (5, 78) = 1.77	P=0.1286	0.58	F (5., 78) = 0.34	P=0.8844	0.37	F (5, 84) = 0.3	P=0.9124
**G x E**	0.52	F (1. 78) = 2.71	P=0.1039	0.25	F (1, 78) = 1.16	P=0.2859	0.31	F (1, 78) = 0.92	P=0.3416	2.11	F (1, 84) = 8.56	**P=0.0044**
**V x G x E**	0.16	F (5, 78) = 0,17	P=0,9735	0.17	F (5, 78) = 0.16	P=0.9772	2.52	F (5, 78) = 1.5	P=0.1994	0.69	F (5, 84) = 0.56	P=0.7300

Table S10. Results of three-way ANOVA testing the effects of frequency of stimulation (from 10 to 100 Hz), genotype (WT *vs.* $TRPV1^{-/-}$) and exercise (sedentary *vs.* trained) on relative force-frequency relationship of EDL and SOL muscles.

	EDL						SOL					
	Male			Female			Male			Female		
**Factor**	**%**	**F (DFn, DFd)**	**P value**	**%**	**F (DFn, DFd)**	**P value**	**%**	**F (DFn, DFd)**	**P value**	**%**	**F (DFn, DFd)**	**P value**
**Frequency (F)**	65.94	F (9, 130) = 67.66	**P<0.0001**	69.17	F (9, 140) = 72.15	**P<0.0001**	62.60	F (9, 130) = 44.95	**P<0.0001**	66.98	F (9, 140) = 41.69	**P<0.0001**
**Genotype (G)**	0.21	F (1, 130) = 1.93	P=0.1669	0.04	F (1, 140) = 0.42	P=0.5207	0.23	F (1, 130) = 1.46	P=0.2290	3.42	F (1, 140) = 19.08	**P<0.0001**
**Exercise (E)**	17.45	F (1, 130) = 161.1	**P<0.0001**	13.37	F (1, 140) = 125.5	**P<0.0001**	10.98	F (1. 130) = 70.95	**P<0.0001**	<0.01	F (1, 140) < 0.01	P=0.9512
**F x G**	0.28	F (9, 130) = 0.29	P=0.9766	0.03	F (9, 140) = 0.03	P>0.9999	0.03	F (9, 130) = 0.02	P>0.9999	0.4	F (9, 140) = 0.25	P=0.9863
**F x E**	1.74	F (9, 130) = 1.78	P=0.0774	1.1	F (9, 140) = 1.15	P=0.3305	3.42	F (9, 130) = 2.46	**P=0.0127**	0.64	F (9, 140) = 0.4	P=0.9329
**G x E**	0.31	F (1, 130) = 2.89	P=0.0917	0.26	F (1, 140) = 2.42	P=0.1221	0.97	F (1, 130) = 6.29	**P=0.0134**	1.7	F (1, 140) = 9.54	**P=0.0024**
**F x G x E**	0.11	F (9, 130) = 0.11	P=0.9993	0.75	F (9, 140) = 0.78	P=0.6365	0.01	F (9, 130) < 0.01	P>0.9999	0.2	F (9, 140) = 0.13	P=0.9989

Table S11. Results of three-way ANOVA testing the effects of genotype (WT *vs.* TRPV1$^{-/-}$), sex (male *vs.* female) and exercise (sedentary *vs.* trained) on relative tetanus amplitude at fatigue, 5 min recovery and 10 min recovery times of EDL muscles.

	**Fatigue**			**5 min recovery**			**10 min recovery**		
**Factor**	**%**	**F (1, 26)**	**P value**	**%**	**F (1, 26)**	**P value**	**%**	**F (1, 26)**	**P value**
**Exercise (E)**	15.60	5.83	**P=0.0231**	15.77	6.19	**P=0.0196**	16.18	6.41	**P=0.0178**
**Sex (S)**	0.13	0.05	P=0.8283	<0.01	<0.01	P=0.9844	0.16	0.06	P=0.8016
**Genotype (G)**	5.98	2.23	P=0.1471	7.33	2.88	P=0.1018	7.26	2.88	P=0.1019
**E x S**	<0.01	<0.01	P=0.9944	0.03	0.01	P=0.9144	0.07	0.03	P=0.8654
**E x G**	0.01	<0.01	P=0.9417	0.02	<0.01	P=0.9304	<0.01	<0.01	P=0.9701
**S x G**	6.22	2.32	P=0.1395	5.23	2.05	P=0.1638	4.77	1.89	P=0.1810
**E x S x G**	0.98	0.36	P=0.5515	3.25	1.28	P=0.2689	4.12	1.63	P=0.2128

Table S12. Results of three-way ANOVA testing the effects of genotype (WT *vs.* TRPV1$^{-/-}$), sex (male *vs.* female) and exercise (sedentary *vs.* trained) on relative tetanus amplitude at fatigue, 5 min recovery and 10 min recovery times of SOL muscles.

	**Fatigue**			**5 min recovery**			**10 min recovery**		
**Factor**	**%**	**F (1, 27)**	**P value**	**%**	**F (1, 27)**	**P value**	**%**	**F (1, 27)**	**P value**
**Exercise (E)**	0.10	0.04	P=0.8494	0.26	0.1	P=0.7573	0.49	0.17	P=0.6804
**Sex (S)**	7.24	2.64	P=0.1158	8.02	3.04	P=0.0926	3	1.05	P=0.3140
**Genotype (G)**	4.05	1.48	P=0.2347	0.3	0.11	P=0.7384	0.04	0.01	P=0.9116
**E x S**	13.78	5.02	**P=0.0334**	14.43	5.47	**P=0.0270**	11.71	4.11	P=0.0526
**E x G**	1.99	0.73	P=0.4015	6.66	2.52	P=0.1238	8.63	3.03	P=0.0932
**S x G**	0.14	0.05	P=0.8254	0.89	0.34	P=0.5659	0.03	0.01	P=0.9151
**E x S x G**	<0.01	<0.01	P=0.9828	<0.01	<0.01	P=0.9973	0.01	<0.01	P=0.9474
